# RNA-seq analysis of *Macrobrachium rosenbergii* hepatopancreas in response to *Vibrio parahaemolyticus* infection

**DOI:** 10.1186/s13099-015-0052-6

**Published:** 2015-03-14

**Authors:** Rama Rao, Ya Bing Zhu, Tahereh Alinejad, Suma Tiruvayipati, Kwai Lin Thong, Jun Wang, Subha Bhassu

**Affiliations:** Genomic Research and Breeding Laboratory and Centre for Research in Biotechnology for Agriculture (CEBAR), Institute of Biological Sciences, Faculty of Science, University of Malaya, 50603 Kuala Lumpur, Malaysia; Beijing Genomics Institute, Shenzhen, 11th Floor, Main Building, Beishan, Industrial Zone, Yantian District, Shenzhen, 518083 China; Microbiology Unit, Institute of Biological Sciences, Faculty of Science, University of Malaya, 50603 Kuala Lumpur, Malaysia

**Keywords:** Transcriptomics, *Macrobrachium rosenbergii*, *Vibrio parahaemolyticus*, *de novo* assembly, Immune genes, Host-pathogen interaction

## Abstract

**Background:**

The Malaysian giant freshwater prawn, *Macrobrachium rosenbergii*, is an economically important crustacean worldwide. However, production of this prawn is facing a serious threat from Vibriosis disease caused by *Vibrio* species such as *Vibrio parahaemolyticus*. Unfortunately, the mechanisms involved in the immune response of this species to bacterial infection are not fully understood. We therefore used a high-throughput deep sequencing technology to investigate the transcriptome and comparative expression profiles of the hepatopancreas from this freshwater prawn infected with *V. parahaemolyticus* to gain an increased understanding of the molecular mechanisms underlying the species’ immune response to this pathogenic bacteria.

**Result:**

A total of 59,122,940 raw reads were obtained from the control group, and 58,385,094 reads from the *Vibrio*-infected group. Via *de novo* assembly by Trinity assembler, 59,050 control unigenes and 73,946 *Vibrio*-infected group unigenes were obtained. By clustering unigenes from both libraries, a total of 64,411 standard unigenes were produced. The standard unigenes were annotated against the NCBI non-redundant, Swiss-Prot, Kyoto Encyclopaedia of Genes and Genome pathway (KEGG) and Orthologous Groups of Proteins (COG) databases, with 19,799 (30.73%), 16,832 (26.13%), 14,706 (22.83%) and 7,856 (12.19%) hits respectively, giving a final total of 22,455 significant hits (34.86% of all unigenes). A Gene Ontology (GO) analysis search using the Blast2GO program resulted in 6,007 unigenes (9.32%) being categorized into 55 functional groups. A differential gene expression analysis produced a total of 14,569 unigenes aberrantly expressed, with 11,446 unigenes significantly up-regulated and 3,103 unigenes significantly down-regulated. The differentially expressed immune genes fall under various processes of the animal immune system.

**Conclusion:**

This study provided an insight into the antibacterial mechanism in *M. rosenbergii* and the role of differentially expressed immune genes in response to *V. parahaemolyticus* infection. Furthermore, this study has generated an abundant list of transcript from *M.rosenbergii* which will provide a fundamental basis for future genomics research in this field.

**Electronic supplementary material:**

The online version of this article (doi:10.1186/s13099-015-0052-6) contains supplementary material, which is available to authorized users.

## Background

The Malaysian giant freshwater prawn, *Macrobrachium rosenbergii* (locally known as ‘udang galah’), belongs to the genus *Macrobrachium,* which is the largest genus of the family *Palaemonidae* [1]. They are found in most inland freshwater areas, including lakes, rivers, swamps, estuarine areas, ponds, canals as well as in irrigation ducts [2]. *M. rosenbergii* spends its adult life in fresh water, but requires brackish water during the initial stages of its life cycle [3]. High demand from the aquaculture industry has led to large-scale farming of this prawn in many countries; the major producers being Bangladesh, Brazil, China, Ecuador, India, Thailand, Taiwan Province of China, and Malaysia [4].

The global production of this prawn had increased to over 200 000 tonnes/year by 2002, and income in Asia alone is now worth US$1 billion per annum [5,6]. In Malaysia, the production of cultured *M. rosenbergii* reached 281 metric tonnes by 1998 [4]. Generally, *M. rosenbergii* is assumed to be less resistant towards diseases than penaeid shrimp [7]. However, with the rise of large-scale high density prawn aquaculture techniques, production of this prawn worldwide is facing a serious threat from fatal diseases caused by nodaviruses and bacteria, particularly from the *Vibrio* species [8,9]. The emergence of these pathogens has had a detrimental impact on the *M. rosenbergii* farming industry, causing considerable economic losses.

*Vibrio* is a Gram-negative halophilic bacterium found abundantly in marine and estuarine environments [10,11]. Among the different species, *Vibrio parahaemolyticus* has emerged as an important pathogen for *M. rosenbergii* [12]. Several other marine shrimps such as *Penaeus monodon, Penaeus japonicas* and *Litopenaeus vannamei* have also been found to be susceptible to *Vibrio* infection [13]. Severe *V. parahaemolyticus* infection in prawns leads to a disease known as ‘Vibriosis’ [14,15]. *M. rosenbergii* suffering from vibriosis may appear black in colour on the carapace, with red discolouration of the exoskeleton and loss of appendages within six days, leading to an 80% mortality rate [12].

Acquiring and establishing knowledge regarding host pathogen interactions is necessary to unlock the pathogenesis of a particular disease. Host pathogen interactions can result in acute and adaptive immune responses against an invader; however, this has been lacking in *M. rosenbergii* [16]. The species defends itself against pathogen invasion using an innate immune system involving the cellular and humoral mechanisms [17,18]. Recently, some progress has been made in analysing the molecular mechanisms of shrimp-pathogen interactions, and several immune genes from shrimp have been discovered such as lectins, antimicrobial peptides, prophenoloxidase and manganese superoxide dismutase, using methods such as suppression subtractive hybridization (SSH) and expressed sequence tags (EST) [19-21]. However, these two methods have been found to be laborious and costly, which limits their use for the production of large-scale transcripts [22].

A cutting edge technology has emerged recently, known as Next Generation Sequencing technology (NGS). Currently, there are four established platforms which uses NGS technology: the Illumina Genome Analyzer, the Roche/454 Genome Sequencer FLX Instrument, and the ABI SOLiD System [23,24]. These platforms have proven versatile and cost-effective tools for advanced research in various genomic areas, such as genome sequencing and re-sequencing, DNA methylation analysis, miRNA expression profiling, and also in non-model organisms as the *de novo* transcriptome sequencing [25]. By using the NGS platform, transcriptome analysis can be performed faster and more easily, because it does not require any bacterial cloning of cDNAs [26]. NGS sequencing has the further advantage of generating greater depth of short reads with minimum error rates [27]. Moreover, it is more reliable and efficient than previous methods in measuring transcriptome composition, revealing RNA expression patterns, and discovering new genes on a larger scale [28]. The superiority of this technology also lies in its sensitivity, which allows the detection of low-abundance transcripts [29].

Previous studies have been performed on whole transcriptome sequencing of the hepatopancreas, gill and muscle tissues of *M. rosenbergii* using the Illumina Genome Analyzer IIx platform (Illumina). They successfully produced a comprehensive transcript data for this freshwater prawn, leading to the discovery of new genes [30]. This present study utilised a similar approach to analyse transcriptome data obtained from the hepatopancreas of *M. rosenbergii* experimentally infected with *V. parahaemolyticus*. The aim was to discover, and determine the role of, immune genes in *M. rosenbergii* involved in *V. parahaemolyticus* infection, which in turn could provide insights into the host-pathogen interactions between these two organisms.

## Material and methods

### *M. rosenbergii* and *V. parahaemolyticus* PCV08-7 challenge

*M. rosenbergii* prawns (5-8 g body weight) purchased from a local hatchery (Kuala Kangsar, Perak, Malaysia) were acclimatized at 28 ± 1°C in aerated and filtered freshwater for one week prior to challenge with *V. parahaemolyticus*. During the challenge experiment, the prawns (n = 10) were intramuscularly injected with 100 μl 1X10^5^ cfu cultured *V. parahaemolyticus* [31] whereas another batch of prawns (n = 10) were injected with 100 μl 2% NaCl (1:10, w/v) solution which serves as negative control group. The hepatopancreas tissues of the prawns were dissected at 12 hours post-infection. The tissues were rapidly frozen in liquid nitrogen and stored at −80°C until total RNA extraction. The 12 hour time point was chosen based on our previous work regarding immune related genes from *M. rosenbergii* in response to pathogen such as viruses showing significant gene expression at this time point [32-35].

### Total RNA extraction and next-generation sequencing

Total RNA (~20 mg) was isolated from both the *V. parahaemolyticus-*challenged and negative control group hepatopancreases. The RNA extraction process was performed by using the Macherey-Nagel NucleoSpin RNA II extraction kit in accordance with the manufacturer’s protocols and stored at −80°C prior to RNA sequencing. The purity and integrity of the RNA was assessed by using the Bioanalyzer 2100 (Agilent technologies, USA). In each group, the total RNA samples were pooled from 10 prawns after which cDNA was synthesized followed by sequencing. The sequencing run was conducted on an Illumina HiSeq™ 2000 platform at the Beijing Genome Institute, Shenzhen, China. The sequencing data constituted 90 bp paired end read data, with ~117 million raw reads.

### Assembly and functional annotation

The raw reads were primarily quality filtered to remove adaptor sequences followed by removal of ambiguous ‘N’ nucleotides (with a ratio of ‘N’ more than 10%) and sequences with a phred quality score of less than 20 before proceeding to *de novo* assembly by using the Trinity software [36]. The Trinity programme assembles the reads into contigs and these contigs were assembled to unigenes. Finally, the TIGR Gene Indices clustering tools (TGICL) [37] with default parameters was applied to cluster the unigenes from both groups which produces non-redundant unigenes.

The non-redundant unigene sequences were aligned to databases which included NCBI non-redundant (Nr), Swissprot [38], Cluster of Orthologous Groups (COG) [39] and Kyoto Encyclopaedia of Genes and Genome (KEGG) [40] using BLASTX [41] with an E-value cut-off of 10^−5^. Gene Ontology (GO) was conducted utilizing default parameters using the BLAST2GO software [42,43]. It was from the above mentioned databases that the gene direction of the unigenes which were annotated and the coding sequence were determined from the BLAST results. The prediction for the coding sequence and the gene direction was performed by ESTscan [44] for those sequences with no defined annotation by using BLAST predicted coding sequence data as the training set.

### Identification of differentially expressed unigenes

The FPKM method (Fragments Per kb per Million fragments) was used to calculate the transcript expression levels [45]. An FDR (false discovery rate) of <0.001 was used as the threshold p-value in multiple tests to judge the degree of differences in gene expression [46]. In a given library when the p-value was less than 0.001 and when the expression level showed greater than two-fold change between two groups genes were considered as differentially expressed.

### Quantitative RT-PCR analysis

We selected seven differentially expressed *M. rosenbergii* unigenes (arginine kinase 1, anti-lipopolysaccharide factor, inhibitor of apoptosis protein, caspase, heat shock protein 21, lectin 1, and NF-kappa B inhibitor alpha) for quantitative RT-PCR analysis (qRT-PCR) to evaluate our Illumina sequencing result. The primer design for the seven unigenes was performed by using Primer3 software http://www.bioinformatics.nl/cgi-bin/primer3plus/primer3plus.cgi/ and listed in Additional file 1. Using 1 μg of RNA, first strand cDNA synthesis was carried out (similar to the sample used for transcriptome sequencing) by using the ImProm-II™ Reverse Transcription System (Promega). The qRT-PCR reaction (20 μl) consisted of a 10 μl TaqMan Universal RT-PCR Master Mix (Applied Biosystems, Foster City, CA, USA), a 1 μl of primers/probe set containing 900 nM of forward reverse primers, a 300 nM probe and 2 μl of template cDNA. The qRT-PCR program was set with an incubation step at 50°C for 2 min, 40 cycles at 95°C for 10 min, 95°C for 15 sec, and 60°C for 1 min, carried out by using Step One Plus Real-Time PCR System® (Applied Biosystems). Similar qRT-PCR cycle profile was applied for the internal control gene, Elongation factor 1-alpha (primer sequences are listed in Additional file 1). The expression level of the seven immune genes were analysed by using the comparative CT method (2 ^-ΔΔCT^ method) [47].

## Results

### Illumina sequencing and assembly

The task of profiling all the immune-related genes involved in *V .parahaemolyticus* infection began with sequencing the two cDNA libraries prepared from pooled mRNAs obtained from the hepatopancreases of the control and infected groups using the Illumina HiSeq™ 2000 platform. A total of 59,122,940 raw reads were obtained from the control group, and 58,385,094 reads from the *Vibrio*-infected group. The raw reads were further filtered to remove adaptor sequences, ambiguous reads and low quality reads, thereby generating 90-bp of 54,708,014 and 54,295,342 clean reads for the control and infected groups respectively ( Q_20_ ~ 98% and percentage of unknown nucleotide is 0%). All sequencing reads were deposited into the Short Read Archive (SRA) of the National Centre for Biotechnology Information (NCBI), and can be accessed under the accession number SRR1424572 for control and SRR1424574 for *Vibrio*-infected ones.

All the clean reads were subjected to *de novo* assembly using the Trinity program which uses three independent software modules – Inchworm, Chrysalis, and Butterfly – applied sequentially to process the huge sequencing data of RNA-seq reads. The assembly of the reads produced 95,645 contigs (with an N_50_ of 467 bp and mean length of 313 bp) for the control group and 123,141 contigs (with an N_50_ of 482 bp and mean length of 318 bp) for *Vibrio*-infected group. These contigs were further assembled into unigenes, producing 59,050 control unigenes (with an N_50_ of 685 bp and mean length of 479 bp) and 73,946 infected group unigenes (with an N_50_ of 829 bp and mean length of 532 bp). The length distribution of control and *Vibrio*-infected contigs and unigenes are shown in Additional file 2. By clustering unigenes from both libraries, a total of 64,411 standard unigenes were produced, with a mean size of 698 bp and an N_50_ of 1137 bp. An overview of the sequencing and assembly is shown in Table 1.

**Table 1:** **Summary of the control and** ***V. parahaemolyticus*** **infected transcriptome sequencing**

	**Control**	***V. parahaemolyticus*** **infected**
Total number of clean reads	54,708,014	54,295,342
Total base pairs (bp)	4,923,721,260	4,886,580,780
Q20 value	97.73%	97.77%
Total number of contigs	95,645	123,141
Mean length of contigs (bp)	313	318
Total number of unigenes	59,050	73,946
Mean length of unigenes (bp)	479	532
NCBI Nr annotated	19,799	
Swiss-Prot annotated	16,832	
KEGG annotated	14,706	
COG annotated	7,856	
GO annotated	6,007	

The standard unigenes were annotated by searching the sequences using BLASTX against the NCBI non-redundant, Swiss-Prot, Kyoto Encyclopaedia of Genes and Genome pathway (KEGG) and Orthologous Groups of Proteins (COG) databases, which produced 19,799 (30.73%), 16,832 (26.13%), 14,706 (22.83%) and 7,856 (12.19%) hits respectively, giving a final total of 22,455 significant hits (34.86% of all unigenes). The size distribution profile for the coding sequences (CDS) and identified proteins are shown in Additional file 2. A Gene Ontology (GO) analysis search using the Blast2GO program resulted in 6,007 unigenes (9.32%) being categorized into 55 functional groups. The unigenes without hits using the BLASTX analysis were subjected to an ESTScan, producing 4,977 unigenes (7.82%) predicted to contain coding sequences. The size distribution of the ESTs and proteins are shown in Additional file 2.

**Figure 1:**
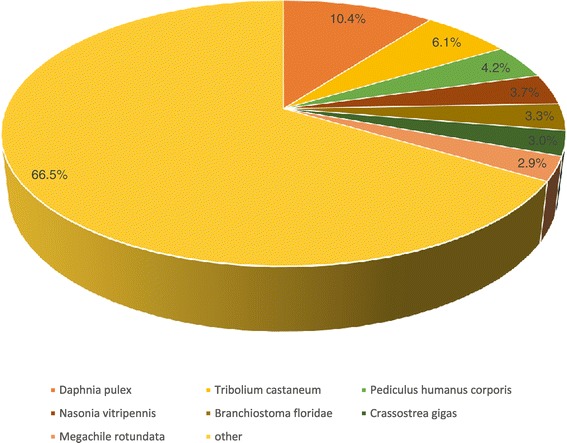
**Species distribution of the BLASTX matches of the transcriptome unigenes.** This figure shows the species distribution of unigene BLASTX matches against the nr protein database (cutoff value E<10^−5^) and the proportions for each species.

The species distribution of the unigenes using the BLASTX results is shown in Figure 1. The *M. rosenbergii* unigenes were matched against *Daphnia pulex* sequences (10.3%), *Tribolium castaneum* (6.1%) and *Pediculus humanus corporis* (4.2%). The unigenes showed a match with those of *D. pulex* and *T. castaneum* probably because of their closer phylogenetic relationship and the availability of vast genomic information. The remaining unigenes (66.5%) which matched were similar to other species due to limited genome information in crustaceans.

### Functional annotation

**Figure 2:**
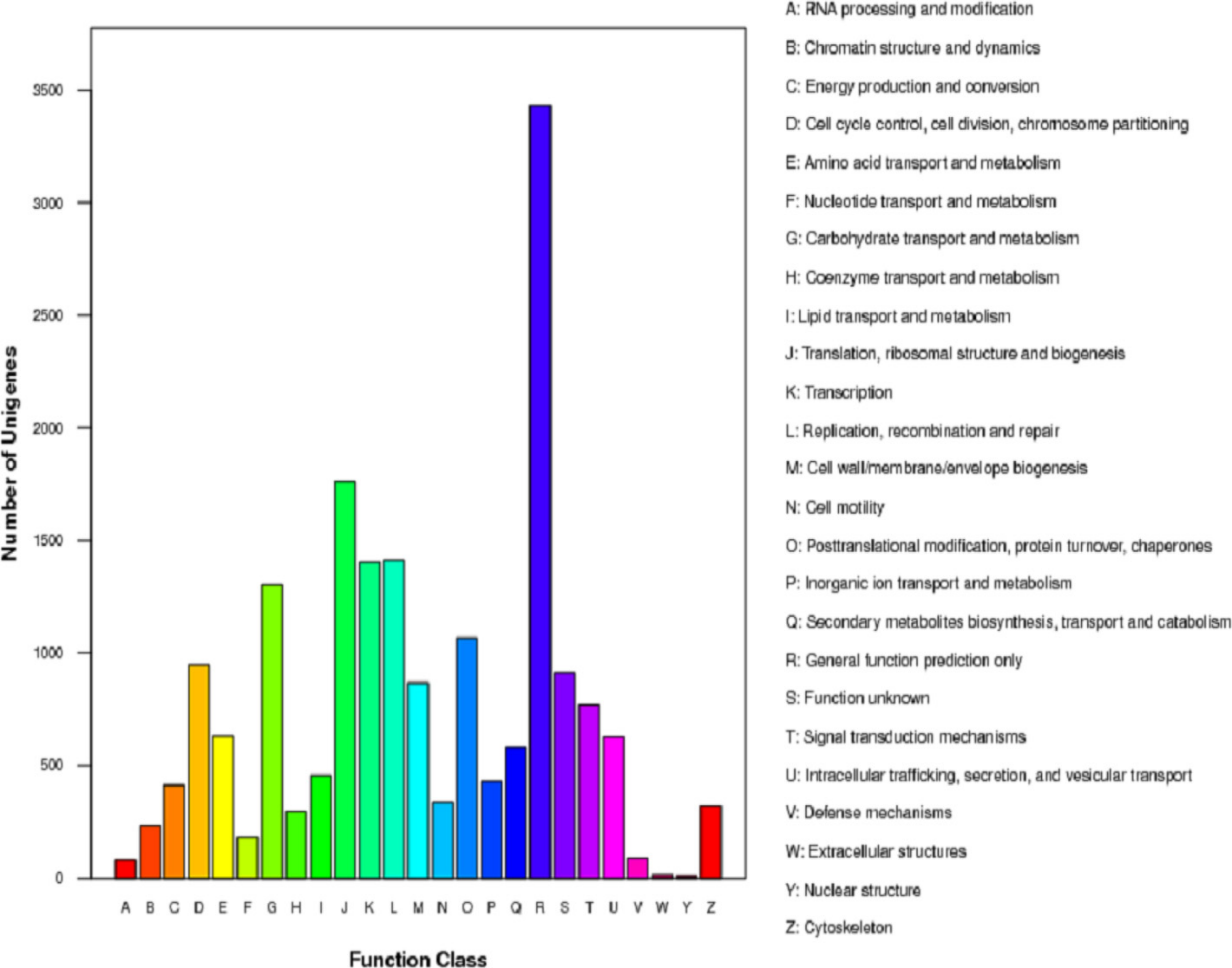
**Histogram presentation of Cluster of Orthologus Groups (COG) classification of 7,856 known protein annotated unigenes.** Each bar represents the number of unigenes classified into each of the 25 COG functional categories.

The standard unigenes were analysed using the COG database to classify them and predict their functions. A total of 7,856 unigenes were assigned to COG classifications and functionally classified into 25 protein families which mainly were involved in cellular structure, biochemistry metabolism, molecular processing, and signal transduction (Figure 2). The cluster predicted for general function (3,431, 43.67%) emerged as the largest group, followed by translation, ribosomal structure, biogenesis cluster (1,760, 22.40%) and the replication, recombination, repair clusters (1,413, 17.98%). The clusters with the lowest number of unigenes were nuclear structure and extracellular structures (<1% in each cluster).

**Figure 3:**
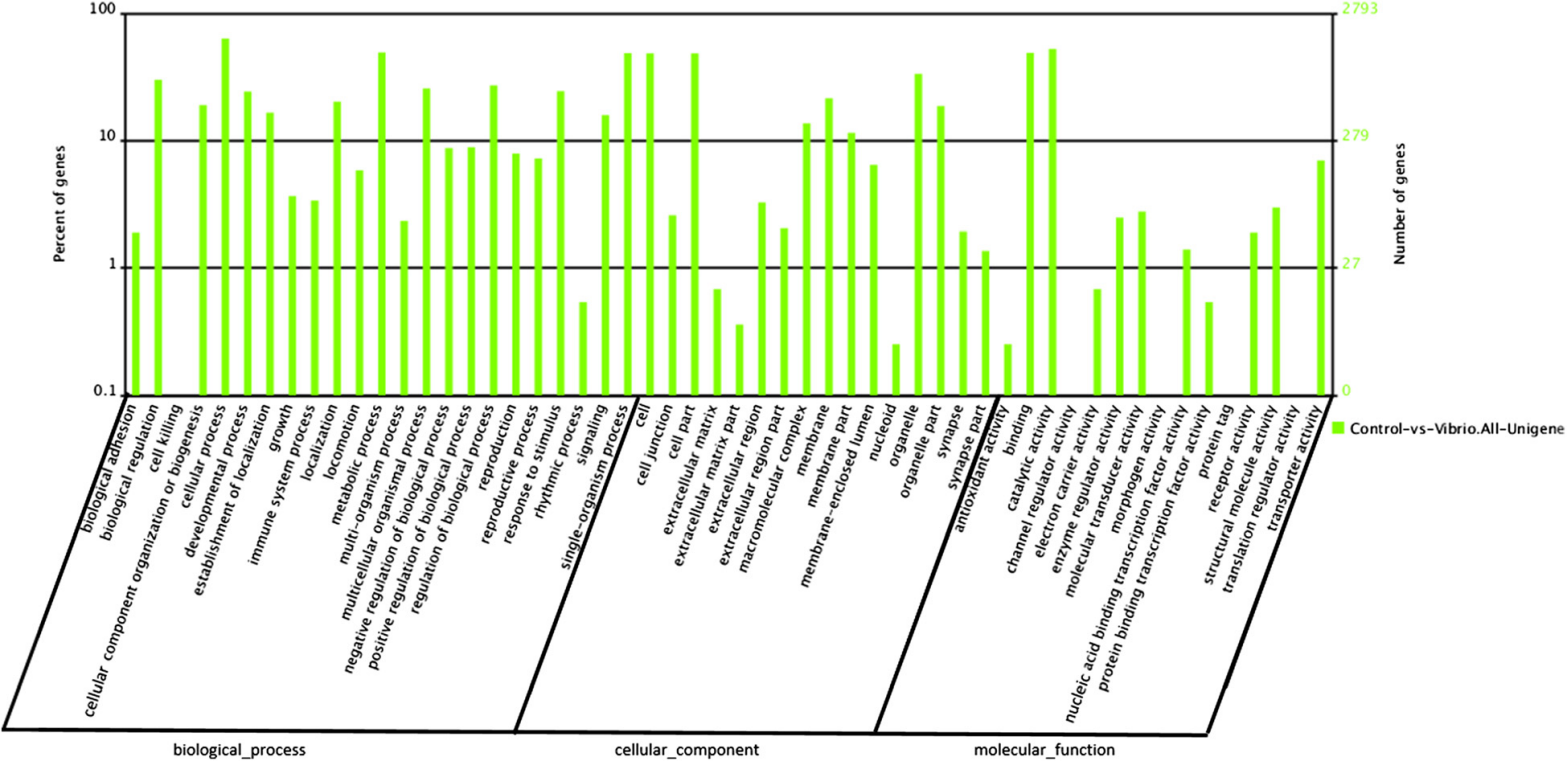
**Gene ontology (GO) classification of the 6,007 protein annotated unigenes.** Unigenes sequences were systematically classified into GO sub-categories under the biological process, cellular component and molecular function gene ontology catalogue system. Each bar represents the relative abundance of unigenes classified under each sub-category.

The standard unigenes with Nr annotations were subjected to Gene Ontology (GO) analysis, which provides a dynamic controlled vocabulary and hierarchical relationship for the representation of information on molecular functions, cellular components and biological processes, allowing a coherent annotation of gene products. The GO annotations produced 6,007 unigenes (biological process: 2,177 unigenes; cellular component: 1,563 unigenes; and molecular function: 2,267 unigenes) which were assigned to 55 GO ontology sub-categories (Figure 3). In the molecular function category, most of the genes fell into the ‘binding’ and ‘catalytic activity’ groups, whereas in the biological process category, the majority of the genes fell into the categories of ‘cellular processes’, ‘metabolic processes’ and ‘single-organism processes’. Finally, in the cellular component category, a high percentage of the genes fell into the ‘cell’ , ‘cell part’ and ‘organelle’ sub-categories.

**Figure 4:**
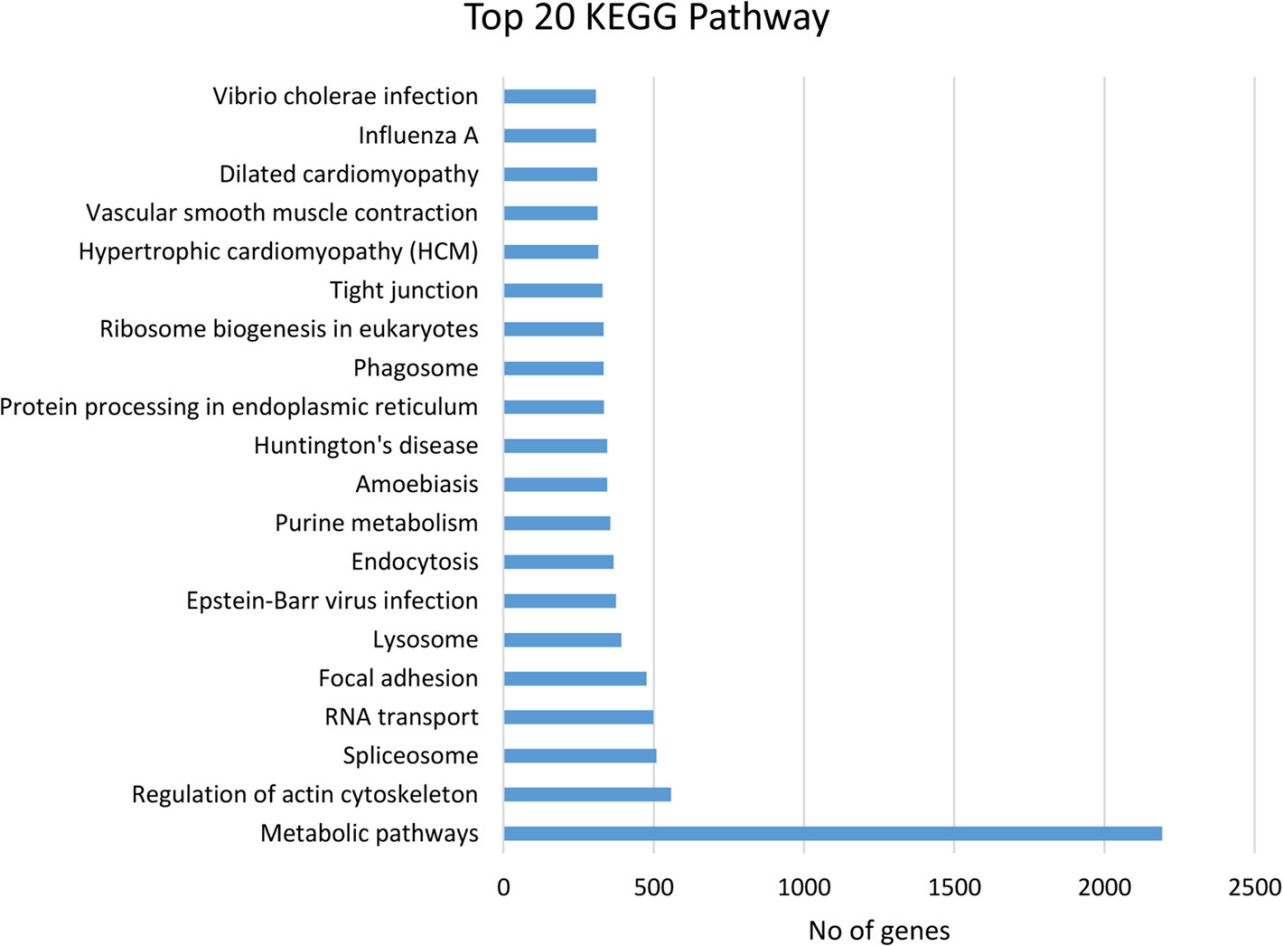
**Top 20 KEGG biological pathway classification histograms for annotated unigenes.**

The Kyoto Encyclopaedia of Genes and Genomes (KEGG) Pathway is a collection of manually drawn pathway maps representing knowledge on molecular interactions and reaction networks. Pathway-based analysis are helpful in identifying biological functions and gene interactions in the pathway. Using the KEGG database, 14,706 unigenes were grouped into 253 pathways. The majority of the unigenes fell into the categories of “metabolic pathways” (2192 members, 14.91%), “regulation of actin cytoskeleton” (557 members, 3.79%), “spliceosome” (509 members, 3.46%), “RNA transport” (498 members, 3.38%) and “focal adhesion” (475 members, 3.22% each). The least represented pathways, with less than 10 unigenes categorized in each pathway, were “biotin metabolism”, “phenylalanine, tyrosine and tryptophan biosynthesis”, “vitamin B6 metabolism”, “lipoic acid metabolism” and “thiamine metabolism”. The top twenty of these KEGG biological pathway classifications are shown in Figure 4.

### Identification of aberrantly expressed genes

**Figure 5:**
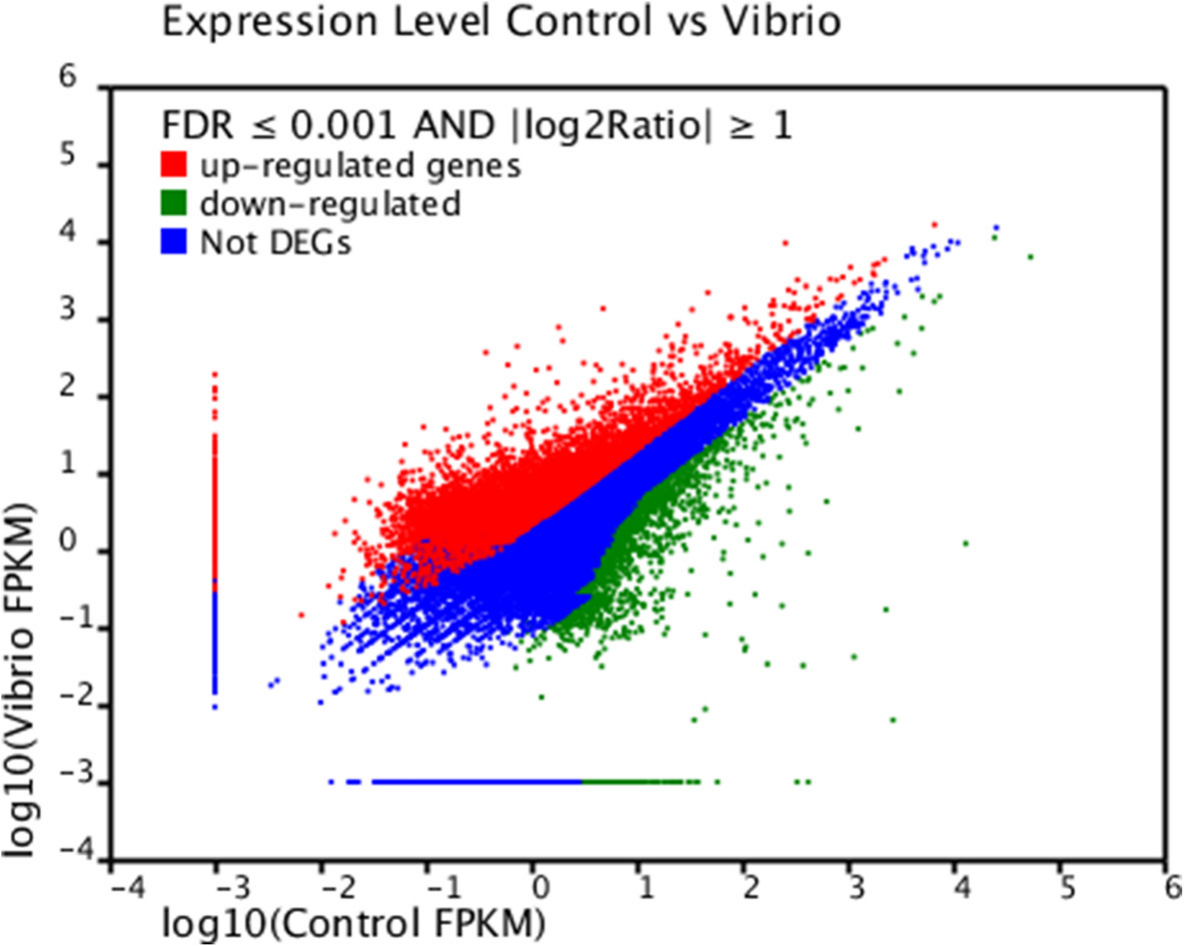
**Digital gene expression between control group and** ***V. parahaemolyticus*** **infected group.** Each point represents a unigene. The x- and y-axis are the log10 of the normalized expression level (FPKM) of unigene between the two groups. Red and green points indicate significant change at the absolute value of log2 (FPKM ratio in two groups) ≥1 and fdr =0.001. Red points indicate up-regulated unigenes and green points indicate down-regulated unigenes in the two groups which its expression level is represented by the y-axis. Blue points indicate insignificant differentially expressed unigenes.

We identified differentially expressed genes between these two groups by comparing the relative transcript abundance in each unigene by using the FPKM method (Fragments Per kb per Million fragments). A total of 14,569 unigenes were found to be aberrantly expressed; 11,446 of these were significantly up-regulated, whereas 3,103 unigenes were significantly down-regulated (Figure 5). The differentially expressed genes were annotated against the NR, Swiss-Prot, GO, COG and KEGG databases by BLASTX with a cut-off E-value of 10^−5^. The annotation analysis is presented in Additional file 3. The 9,469 (65%) unigenes containing low sequence homology to known sequences in public databases could represent non-coding RNA, misassembled unigenes or unknown genes of *M. rosenbergii* which responded to *V. parahaemolyticus*-infection.

**Figure 6:**
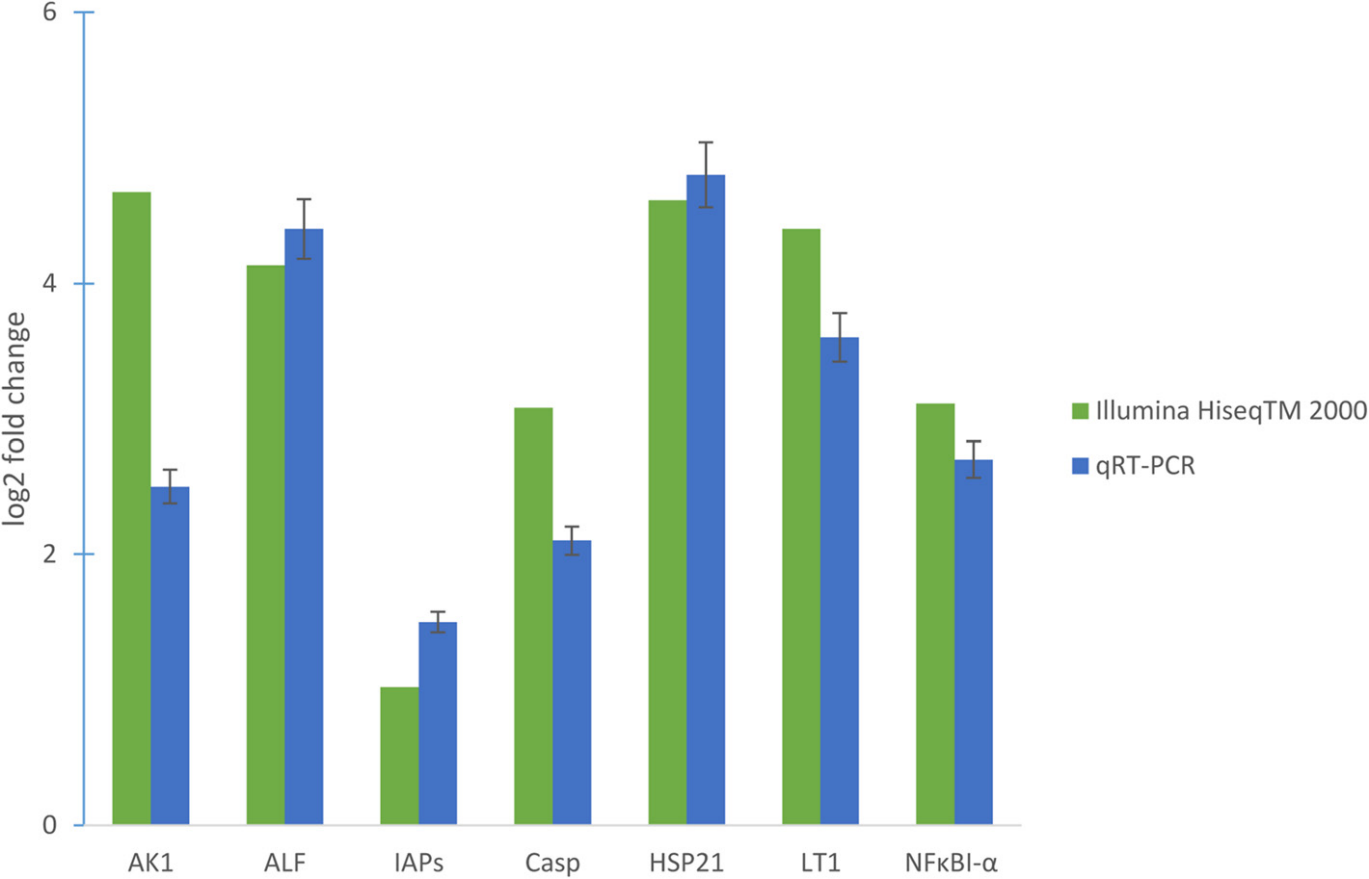
**Comparison of the expression profiles of selected genes as determined by Illumina Hiseq** ^**TM**^ **2000 sequencing (green) and qRT-PCR (blue).** Target gene abbreviations are as follows: AK1 - arginine kinase, ALF- anti-lipopolysaccharide factor, IAPs- apoptosis inhibitor, Casp- caspase, HSP21- heat shock protein 21, LT1- lectin 1, NFκBI-α - NF-kappa B inhibitor alpha. Error bars indicated standard deviations of averages from three replicates.

For validation of the Illumina sequencing result, seven unigenes were chosen randomly for quantitative real time-PCR (qRT-PCR) analysis. The qRT-PCR results showed similar trends for all the genes to the sequencing data (Figure 6). For example, based on the Illumina sequencing analysis, arginine kinase 1, anti-lipopolysaccharide factor, inhibitor of apoptosis protein, caspase, heat shock protein 21, lectin 1 and NF-kappa B inhibitor alpha were up-regulated 4.67, 4.13, 1.02, 3.08, 4.61, 4.4 and 3.11 log2-fold respectively; and the same elements showed 2.5, 4.4, 1.5, 2.1, 4.8, 3.6 and 2.7 log2-fold change respectively in the qRT-PCR analysis. While the results from these two analyses did not match perfectly, perhaps due to sequencing biases, the qRT-PCR analysis broadly confirmed the direction of change obtained from the Illumina sequencing analysis.

### Potential immune-related genes involved in *M. rosenbergii* immune response

Many of the aberrantly expressed genes found in the *V. parahaemolyticus*-infected groups compared to the control group are known to belong to various processes clustered under the animal immune system (Table 2). These immune genes are grouped into 11 functions, including antimicrobial proteins, proteases and proteinases, signal transduction, blood clotting system, cell death, cytoskeletal, heat shock proteins, oxidative stress, pathogen recognition immune receptors, prophenoloxidase system and other immune genes. The role of these groups is described in more detail in the next (Discussion) section.

**Table 2:** **Candidate genes involved in** ***M. rosenbergii*** **immune response against** ***V. parahaemolyticus***

**Category or gene id**	**Homologues function**	**Species**	FC*
**Antimicrobial**			
Unigene4120_all	Anti-lipopolysaccharide factor	*Macrobrachium rosenbergii*	4.13
Unigene23546_all	Anti-lipopolysaccharide factor 2	*Macrobrachium rosenbergii*	2.84
CL1276.Contig1_all	Anti-lipopolysaccharide factor 3	*Macrobrachium rosenbergii*	2.15
Unigene37309_all	Crustin	*Macrobrachium rosenbergii*	5.13
Unigene26338_all	Lysozyme	*Portunus trituberculatus*	1.92
Unigene13073_all	NF-kappa B inhibitor alpha	*Macrobrachium rosenbergii*	3.11
**Blood clotting system**			
Unigene13048_all	Clottable protein	*Marsupenaeus japonicus*	1.42
Unigene34308_all	Transglutaminase	*Macrobrachium rosenbergii*	11.34
Unigene36567_all	Proclotting enzyme	*Harpegnathos saltator*	11.22
Unigene41253_all	Coagulation factor XII	*Hageman factor*	10.61
**PRPs**			
Unigene10978_all	Lectin 1	*Macrobrachium rosenbergii*	4.4
CL4516.Contig1_all	Lectin 2	*Macrobrachium rosenbergii*	1.05
Unigene7825_all	Lectin 3	*Macrobrachium rosenbergii*	2.54
CL3039.Contig4_all	Lectin 4	*Macrobrachium rosenbergii*	4.55
Unigene9635_all	C-type lectin	*Penaeus monodon*	1.14
CL1600.Contig1_all	C-type lectin-2	*Litopenaeus vannamei*	1.69
CL5230.Contig3_all	C-type lectin 5	*Fenneropenaeus chinensis*	2
Unigene1391_all	Hemolectin	*Papilio xuthus*	5.81
Unigene25701_all	M-type lectin	*Marsupenaeus japonicus*	1.69
Unigene23215_all	Perlucin-like protein	*Mytilus galloprovincialis*	−1.92
CL1075.Contig4_all	Tachylectin	*Macrobrachium rosenbergii*	−1.71
Unigene28944_all	Ficolin	*Branchiostoma floridae*	1.31
Unigene19793_all	Lectin B isoform 2	*Marsupenaeus japonicus*	3.55
Unigene37041_all	Mannose-binding protein	*Procambarus clarkii*	11.69
CL1124.Contig1_all	Glucan pattern-recognition lipoprotein	*Fenneropenaeus chinensis*	1.12
Unigene23671_all	lipopolysaccharide and beta-1,3-glucan binding protein	*Macrobrachium rosenbergii*	1.71
**Proteinases and proteinases inhibitors**			
Unigene1509_all	Cathepsin B	*Pandalus borealis*	1.87
Unigene22859_all	Cathepsin C	*Fenneropenaeus chinensis*	2.33
CL1587.Contig2_all	Cathepsin D	*Penaeus monodon*	−1.56
Unigene26111_all	Cathepsin L	*Pandalus borealis*	1.41
CL5824.Contig1_all	Serpin serine protease inhibitor	*Fenneropenaeus chinensis*	12.24
CL6038.Contig1_all	26S protease regulatory	*Nasonia vitripennis*	1.44
Unigene12869_all	Alpha-2-macroglobulin	*Macrobrachium rosenbergii*	5.09
CL2365.Contig1_all	Caspase	*Marsupenaeus japonicus*	1.04
Unigene13293_all	Astacin	*Strongylocentrotus purpuratus*	1.64
Unigene13633_all	Serine protease	*Fenneropenaeus chinensis*	3.08
Unigene17259_all	Caspase 8	*Ictalurus punctatus*	2.41
CL2565.Contig1_all	Serine proteinase inhibitor 6	*Penaeus monodon*	4.51
CL1118.Contig1_all	Serpin B	*Marsupenaeus japonicus*	7.02
Unigene26757_all	Hemocyte kazal-type proteinase inhibitor	*Penaeus monodon*	3.87
CL1127.Contig2_all	Kazal-type serine proteinase inhibitor 4	*Procambarus clarkii*	−6.01
CL5487.Contig2_all	Aminopeptidase N	*Camponotus floridanus*	1.4
Unigene5621_all	Masquerade-like serine proteinase-like protein 2	*Penaeus monodon*	5.39
Unigene21512_all	Serine proteinase inhibitor	*Macrobrachium rosenbergii*	6.91
Unigene28987_all	CUB-serine protease	*Panulirus argus*	2.92
**Heat shock proteins**			
Unigene3736_all	Heat shock protein 21	*Macrobrachium rosenbergii*	4.61
Unigene23034_all	Heat shock protein 40	*Frankliniella occidentalis*	1.8
Unigene14757_all	Heat shock protein 70	*Portunus trituberculatus*	2.26
CL4309.Contig3_all	Heat shock protein 90	*Scylla paramamosain*	1.93
Unigene16858_all	Small heat shock protein	*Fenneropenaeus chinensis*	2.41
**Oxidative stress**			
CL353.Contig2_all	Glutathione S transferase	*Procambarus clarkii*	1.01
Unigene19405_all	Cu/Zn superoxide dismutase	*Bombyx mori*	1.61
CL5590.Contig3_all	Glutamine synthetase	*Panulirus argus*	−3.03
CL559.Contig1_all	Farnesoic acid O-methyltransferase	*Nilaparvata lugens*	1.81
CL2477.Contig1	Catalase	*Litopenaeus vannamei*	1.6
CL2787.Contig2	Thioredoxin reductase	*Branchiostoma floridae*	1.09
**Cytoskeletal**			
Unigene10471	Chitinase	*Trichomonas vaginalis*	6.16
CL6026.Contig5_all	Actin	*Marsupenaeus japonicus*	2.92
Unigene13021_all	Calponin	*Chironomus riparius*	2.32
CL5710.Contig1_all	Profilin	*Fenneropenaeus chinensis*	1.45
CL461.Contig8_all	Tubulin	*Homarus americanus*	1.39
Unigene19173_all	Beta-integrin	*Fenneropenaeus chinensis*	2.78
CL2046.Contig1_all	Integrin	*Litopenaeus vannamei*	3.03
**Signal transduction**			
***Toll pathway***			
CL1915.Contig5_all	Toll interacting protein	*Marsupenaeus japonicus*	1.73
CL5402.Contig1_all	Toll receptor 2	*Marsupenaeus japonicus*	−2.46
Unigene25507_all	Toll-like receptor 3	*Daphnia pulex*	2.59
CL2421.Contig1_all	Toll-like receptor 8	*Ixodes scapulari*	1.48
Unigene25799_all	Myd88 protein	*Artemia sinica*	3.25
Unigene9776_all	Caspase	*Fenneropenaeus merguiensis*	3.08
Unigene14481_all	Dorsal	*Fenneropenaeus chinensis*	3.19
***Wnt signaling pathway***			
Unigene36938_all	Lipoprotein receptor	*Callinectes sapidus*	10.94
Unigene15960_all	Prickle	*Saccoglossus kowalevskii*	1.71
Unigene15970_all	Low-density lipoprotein receptor-related protein 6	*Daphnia pulex*	2.05
Unigene12737_all	Glypican 4	*Daphnia pulex*	1.14
Unigene25938_all	Secreted frizzled-related protein 5	*Daphnia pulex*	1.82
***MAPK signaling pathway***			
Unigene26817_all	Mitogen-activated protein kinase kinase kinase 1	*Oryctolagus cuniculus*	2.79
Unigene110_all	Mitogen-activated protein kinase 8 interacting protein 3	*Bombus terrestris*	2.94
CL726.Contig3_all	Mitogen-activated protein kinase kinase kinase kinase 4	*Bombus terrestris*	1.65
CL3174.Contig1_all	Mitogen-activated protein kinase kinase kinase 7	*Danaus plexippus*	1.41
Unigene29569_all	Mitogen-activated protein kinase kinase kinase 13	*Bombus terrestris*	4.03
CL4432.Contig2_all	Max protein	*Daphnia pulex*	−3.11
Unigene14757_all	Heat shock protein 70	*Portunus trituberculatus*	2.26
Unigene3873_all	Protein phosphatase 5	*Bombus terrestris*	1.21
Unigene102_all	Map kinase-interacting serine/threonine	*Scylla paramamosain*	2.48
Unigene11596_all	Raf homolog serine/threonine-protein kinase phl-like	*Nasonia vitripennis*	2.41
***JAK/STAT pathway***			
Unigene25562_all	STAT long form	*Penaeus monodon*	2.84
Unigene25762_all	Signal transducing adaptor molecule	*Megachile rotundata*	1.98
Unigene16474_all	Tumor susceptibility gene 101 protein	*Harpegnathos saltator*	2.46
CL1133.Contig6_all	Domeless	*Tribolium castaneum*	2.94
Unigene28876_all	Thyroid peroxidase	*Pediculus humanus corporis*	3.66
***Other signal transduction genes***			
CL1409.Contig1_all	Innexin 3	*Cancer borealis*	10.29
Unigene30035_all	cAMP-dependent protein kinase type II regulatory subunit	*Camponotus floridanus*	1.05
CL3406.Contig2_all	Casein kinase II subunit alpha	*Danaus plexippus*	1.75
CL4247.Contig1_all	Rab-protein 14	*Tribolium castaneum*	1.85
Unigene37520_all	Afadin	*Pediculus humanus corporis*	2.89
Unigene34256_all	TBC1 domain family member 10B	*Crassostrea gigas*	11.52
Unigene33128_all	REM2- and Rab-like small GTPase 1-like	*Cavia porcellus*	3.21
**ProPO system**			
Unigene12734_all	Prophenoloxidase	*Macrobrachium rosenbergii*	3.15
Unigene7353_all	Prophenoloxidase activating factor	*Fenneropenaeus chinensis*	3.79
Unigene18337_all	Prophenoloxide activating enzyme III	*Macrobrachium rosenbergii*	4.99
**Cell death**			
Unigene2555_all	Beclin 1	*Megachile rotundata*	11.89
CL1843.Contig2_all	ALG-2 interacting protein x *Penaeus monodon*	*Penaeus monodon*	3.2
Unigene14045_all	Program cell death 5-like	*Penaeus monodon*	1.61
CL2477.Contig1_all	Catalase	*Litopenaeus vannamei*	1.6
Unigene3163_all	inhibitor of apoptosis protein	*Litopenaeus vannamei*	1.02
Unigene9776_all	Caspase	*Fenneropenaeus merguiensis*	3.08
Unigene36052_all	DNA fragmentation factor subunit beta	*Danio rerio*	3.26
**Other immune genes**			
CL4920.Contig1_all	Calmodulin	*Procambarus clarkii*	1.92
CL1939.Contig4_all	Ferritin	*Litopenaeus vannamei*	−2.87
CL4071.Contig2_all	Peritrophin	*Macrobrachium nipponense*	−2.18
Unigene10835_all	Selenoprotein W	*Cricetulus griseus*	−1.98
Unigene26739_all	Metallothionein I	*Macrobrachium rosenbergii*	−2.75
Unigene29279_all	Selenoprotein L	*Ciona intestinalis*	1.06
CL6324.Contig2_all	Arginine kinase 1	*Macrobrachium rosenbergii*	4.67
CL5294.Contig2_all	Hemocyanin	*Macrobrachium nipponense*	1.21
Unigene26326_all	Tetraspanin-like protein CD9	*Fenneropenaeus chinensis*	1.02
CL4316.Contig1_all	Crustacyanin-like lipocalin	*Macrobrachium rosenbergii*	−4.12
CL6297.Contig2_all	E cadherin	*Tenebrio molitor*	3.19
CL2339.Contig2_all	Adenosine deaminase	*Caligus rogercresseyi*	−1.35
CL3196.Contig2_all	Calnexin	*Penaeus monodon*	1.57
CL2701.Contig1_all	Ubiquitin-conjugating enzyme	*Macrobrachium nipponense*	1.33

*Fold changes (Log2 ratio) in gene expression. PRPs- pattern recognition proteins, ProPO- prophenoloxidase.

## Discussion

Apart from viral diseases, *Vibrio* infections causing Vibriosis is another factor hindering the shrimp aquaculture industry worldwide [9]. This fatal disease has contributed to mass mortality and severe economic losses in India, Thailand, Philippines, Japan, Ecuador, Peru, Colombia and Central America [13]. Knowledge about the interaction between *M. rosenbergii* and *Vibrio* species is in its infancy, and in-depth study is urgently needed to address this issue. Discovery of the molecular mechanisms surrounding the innate immune system against *Vibrio* infection in freshwater prawns should be beneficial to both scientific research and the aquaculture industry. Identifying and quantifying immune-related gene expression on a large scale is a promising method to investigate the host response against pathogens and provide a platform for further studies in this area.

Microarray and EST analyses have long been used to study the molecular mechanisms underlying the innate immune system and to identify genes aberrantly expressed during infection [48-50]. However, the most recent NGS platforms such as the Illumina HiSeq™ 2000 appear much better at quantifying transcripts expressed at low levels than microarrays or EST analysis [29]. This is because this revolutionary technique verifies direct transcript profiling without compromise or bias, unlike previous methods [27]. Furthermore, this technology has been successfully used in transcriptome profiling studies on non-model organisms where there is no complete genome database [51-53]. The introduction of NGS technology has led to various studies on host-viral interactions in shrimps to identify potential immune-related genes [54-56] – but not so far on the interaction between the freshwater prawn and *Vibrio* species. To our knowledge, this study could be the very first to use the Illumina HiSeq™ 2000 platform to explore the immune-related gene response in *M. rosenbergii* against *V. parahaemolyticus*.

Taking advantage of the Illumina HiSeq™ 2000 platform’s capability to sequence with a high throughput data providing more candidate genes, the total RNA extracted at the 12th hour time point from a pool of control and infected hepatopancreases was sequenced and assembled using the Trinity assembler. The overall analysis yielded 14,549 differentially expressed unigenes, with 11,446 unigenes significantly up-regulated and 3,103 unigenes significantly down-regulated. The sequencing data analyses obtained clearly showed a significant impact of *V. parahaemolyticus* infection on the *M. rosenbergii* transcriptome.

*M. rosenbergii* possesses an innate immune system, consisting of cellular and humoral components which work individually or cooperatively to protect the species from invading pathogens such as *V. parahaemolyticus* [17,18]. This immune response is activated when the animal detects the invading pathogen through pattern recognition proteins (PRPs) [57]. Two important PRPs molecules identified in shrimp are lectins and the beta-1,3- glucan binding protein (LGBP) [58-61]. Besides pathogen recognition, lectins are also involved in phagocytosis through opsonisation in crustaceans [62]. Our data showed that challenge by *V. parahaemolyticus* greatly affects the expression of PRPs, as observed in previous studies using different *Vibrio* strains [63-67]. The activation of LGBP molecules triggers the melanisation process, a prophenoloxidase-activating system (proPO-AS) which is an enzymatic cascade involving several enzymes, including the key enzyme phenoloxidase (PO) [68-70]. The active PO converts phenols into quinones. These build a non-specific crosslink between neighbouring molecules to form melanin, which provides defence against invading microorganisms [71]. Increased activity of prophenoloxidase against *Vibrio* species has also been noted in *Fenneropenaeus indicus* [72], *L. vannamei* [73] and *P. monodon* [74].

Antimicrobial peptides (AMPs) play a pivotal role in killing or clearing infected pathogens, especially *Vibrio* species [75]. Notable shrimp AMPs, such as penaeidins, lysozymes, crustins, anti-lipopolysaccharide factors (ALFs) and stylicins have been identified and characterized previously in shrimps [76-80]. However, only four types of AMPs – lysozyme, crustin, NF-kappa B inhibitor alpha and ALF (3 isoforms) – were detected in our transcriptome data and found to be highly expressed. The up-regulation of these AMPs correlated with previous studies showing their antimicrobial properties against *Vibrio* species and other bacteria [81-84]. Blood clotting is vital in crustaceans to prevent excess blood loss from a wound and prevent micro-organisms from invading the wound [85]. We found four molecules of the blood clotting system – transglutaminase, clottable protein, proclotting enzyme and coagulation factor XII – to be highly induced in our transcriptome data after challenge by *V. parahaemolyticus*. A similar expression of these molecules after bacterial challenge has been reported in previous studies [72,86,87].

Stress conditions such as bacterial infections lead to an accumulation of reactive oxygen species (ROS) in a cell [88]. Increased levels of ROS causes oxidative damage to important cellular macromolecules (lipids, proteins, carbohydrates and nucleotides) which are components of the membranes, cellular enzymes and DNA [89]. In order to restrict the production of ROS, antioxidant genes are activated to produce antioxidant enzymes which eliminate ROS. Several antioxidant enzymes have been isolated and characterised in the penaeid shrimp in previous studies [90-92]. In this study, we found six antioxidant unigenes to be over-expressed after *V. parahaemolyticus* challenge – the exception being glutamine synthetase. High expression levels of these genes have similarly been observed in other shrimps and scallops after *Vibrio* challenge [93-96]. The up-regulation of actin and tubulin genes play a crucial role in a wide range of cellular functions such as nodule formation, phagocytosis, encapsulation, as well as cell shape change, cell motility and adhesion, all of which may aid in clearing the pathogen [97].

Heat shock proteins (HSPs) are highly generated when induced by stress. They are known to play a major role in protein folding, the protection of proteins from denaturation or aggregation, and aiding protein transport through membrane channels [98,99]. In addition to molecular chaperones, HSPs have been reported to play important roles in innate immune responses, and have been well studied in crustaceans [100-102]. In this study, we noted higher levels of expression of all heat shock proteins in *M. rosenbergii* when challenged by *V. parahaemolyticus*. The increased expression of these HSPs is in line with previous reports, which tends to confirm the important role of these proteins in protecting this species from the stress induced by *Vibrio* challenge [97,103,104]. The general higher expression of proteinases and their inhibitors was to be expected in our data, as these are known to modulate elements of the innate immune system such as haemolymph coagulation, antimicrobial peptide synthesis, cell adhesion, and melanisation [105].

Bacteria like *Vibrio* are known to induce cell apoptosis through a variety of mechanisms such as pore-forming proteins, secretion of protein synthesis inhibitors, molecules activating the endogenous death machinery in an infected cell, lipopolysaccharides, and other superantigens [106]. Increased levels of apoptosis contribute to the degradation of DNA and RNA, which may contribute to shrimp mortality [107]. In our transcriptome data, an up-regulation of genes involved in apoptosis was observed, similar to the trends reported in previous studies [74,108,109]. Apoptosis may also serve as host defence against bacterium by allowing other healthy cells to phagocytise apoptotic bodies containing bacteria from target cells, effectively clearing the pathogen [110].

The signalling pathways involved in the *M. rosenbergii* innate immune response against *V. parahaemolyticus* were observed to be highly induced in our transcriptome data. The Toll protein initially identified in *Drosophila* had been reported to play a key role in the anti-fungal and anti-Gram-positive bacterial responses of flies in the Toll pathway [111]. Other Toll components have also been found to be activated in penaeid shrimps when challenged with *Vibrio* species [109,112,113]. In *Caenorhabditis elegans*, the mitogen-activated protein kinase (MAPK) pathways are transcriptionally up-regulated by the pore-forming toxin released by *Bacillus thuringiensis*, which provide a cellular defence against this toxin [114]. This could explain the higher expression of this signalling pathway in our data, as *V. parahaemolyticus* is known to release a thermostable direct haemolysin, which is a pore-forming toxin [115]. The Janus kinase (JAK) and signal transducer and activator of transcription (STAT) pathways have been reported to be activated when *Fenneropenaeus chinensis* is challenged with *Vibrio anguillarum,* which suggest that these pathways are important for immune responses against bacteria [116]. In addition, Rab-related proteins have been reported to regulate the hemocytic phagocytosis of bacteria in *Marsupenaeus japonicas* [117].

Several genes in the other immune gene group were found to be aberrantly expressed in our transcriptome data. Calmodulin, which plays an important role in calcium-dependent signal transduction pathways, was over-expressed in our data – as was the case in *L. vannamei* when challenged with *V. parahaemolyticus* [118]. Ferritin, an iron storage protein crucial for the metabolism of iron and maintaining iron homeostasis in a cell, was found to be down-regulated. The reduced expression of this gene could possibly lead to prawn mortality, as increased expression of this gene has been found to protect *P. monodon* from *Vibrio harveyi* [119]. Arginine kinase (AK), a phosphagen kinase in the invertebrate energy metabolism, has previously been reported to play an immune role against viral infection [32]. However, we observed a higher expression of this gene in *M. rosenbergii* after challenge by *V. parahaemolyticus,* which could suggest that AK plays a similar role in bacterial infection. Haemocyanin, an important immune gene in crustaceans, is involved in prophenoloxidase activity [120]. It has antiviral properties against WSSV [121], and its increased expression in our transcriptome data tends to bear out its importance as a defence molecule against challenge by *V. parahaemolyticus*. Finally, metallothioneins, a metal-binding protein, was found to be highly expressed in our transcriptome data. The increased expression of this gene was to be expected, as it is known as a scavenger of reactive oxygen intermediates and generally shows higher expression levels during immune responses in invertebrates against pathogens [49].

## Conclusion

We utilised the Illumina HiSeq™ 2000 platform and Trinity assembler package to perform a *de novo* transcriptome profiling of the hepatopancreases isolated from *M. rosenbergii* challenged with *V. parahaemolyticus.* The differential expression analysis between *V. parahaemolyticus*-infected and control groups revealed significant differences in the gene expression with 11,446 unigenes found to be significantly up-regulated and 3,103 unigenes observed to be significantly down-regulated. This study provided a valuable insight into antibacterial mechanisms of freshwater prawn against *V. parahaemolyticus* with majority of the differentially expressed unigenes were grouped into 11 animal immune system categories. Furthermore, this study has generated an abundant list of transcript from *M.rosenbergii* which will provide a fundamental basis for future genomics research in this field.

## Additional files

Additional file 1: Table S1. List of genes, primers and probes used in real-time TaqMan PCR assay. Additional file 2: Figure S1. Overview of the control and *V. parahaemolyticus* infected transcriptome assembly. **(A)** The size distribution of the contigs obtained from our *denovo* assembly of high-quality clean reads. **(B)** The size distribution of the unigenes produced from further assembly of contigs (i.e., contig joining, gap filling, and scaffold clustering). **(C)** Size distributions of the coding sequences (CDS) and identified proteins and **(D)** Size distributions of the ESTs and proteins obtained from the ESTScan results. For unigene CDS that had no hits in the databases (Nr, SwissProt, KEGG and COG), the BLAST results were subjected to ESTScans and then translated into peptide sequences. Additional file 3: Table S2. List of differentially expressed genes with Nr, Nt, Swissprot, GO, COG, KEGG.

## References

[1] De Grave S, Cai Y, Anker A. Global diversity of shrimps (Crustacea: Decapoda: Caridea) in freshwater. Hydrobiologia 2008 (595):287–293.

[2] New MB. Farming freshwater prawns: a manual for the culture of the giant river prawn (Macrobrachium rosenbergii): Food & Agriculture Org. 2002;(148):1-11.

[3] Wowor D, Muthu V, Meier R, Balke M, Cai Y, Ng PK (2009). Evolution of life history traits in Asian freshwater prawns of the genus Macrobrachium(Crustacea: Decapoda: Palaemonidae) based on multilocus molecular phylogenetic analysis. Mol Phylogenet Evol.

[4] New MB. History and global status of freshwater prawn farming. *Freshwater Prawns: Biology and Farming* 2009(194):16–40.

[5] Schwantes VS, Diana JS, Yi Y (2009). Social, economic, and production characteristics of giant river prawn Macrobrachium rosenbergii culture in Thailand. Aquaculture.

[6] New MB (2005). Freshwater prawn farming: global status, recent research and a glance at the future. Aquacult Res.

[7] Nash G, Chinabut S, Limsuwan C (1987). Idiopathic muscle necrosis in the freshwater prawn, Macrobrachium rosenbergii de Man, cultured in Thailand. J Fish Dis.

[8] Bonami J-R, Sri Widada J (2011). Viral diseases of the giant fresh water prawn Macrobrachium rosenbergii: a review. J Invertebr Pathol.

[9] Tonguthai K (1995). Diseases of the freshwater prawn, Macrobrachium rosenbergii, The Aquat. Anim Health Res Inst Newsl.

[10] Ramesh A, Loganathan B, Venkateswaran K (1990). Ecological dynamics of marine luminous bacteria. J Basic Microbiol.

[11] Thompson FL, Iida T, Swings J (2004). Biodiversity of vibrios. Microbiol Mol Biol Rev.

[12] Khuntia CP, Das BK, Samantaray BR, Samal SK, Mishra BK (2008). Characterization and pathogenicity studies of Vibrio parahaemolyticus isolated from diseased freshwater prawn, Macrobrachium rosenbergii (de Man). Aquacult Res.

[13] Lightner DV (1996). A handbook of shrimp pathology and diagnostic procedures for diseases of cultured penaeid shrimp.

[14] Ruangpan L, Kitao T (1991). Vibrio bacteria isolated from black tiger shrimp, Penaeus monodon Fabricius. J Fish Dis.

[15] Xu B, Xu H, Ji W, Shi J (1994). Pathogens and pathogenicity to Penaeus orientalis Kishinouye. Acta Oceanol Sinic.

[16] Kimbrell DA, Beutler B (2001). The evolution and genetics of innate immunity. Nat Rev Genet.

[17] Jiravanichpaisal P, Lee BL, Söderhäll K (2006). Cell-mediated immunity in arthropods: hematopoiesis, coagulation, melanization and opsonization. Immunobiology.

[18] Young Lee S, Söderhäll K (2002). Early events in crustacean innate immunity. Fish Shellfish Immunol.

[19] Zhao Z-Y, Yin Z-X, Weng S-P, Guan H-J, Li S-D, Xing K (2007). Profiling of differentially expressed genes in hepatopancreas of white spot syndrome virus-resistant shrimp ( Litopenaeus vannamei) by suppression subtractive hybridisation. Fish Shellfish Immunol.

[20] Pan D, He N, Yang Z, Liu H, Xu X (2005). Differential gene expression profile in hepatopancreas of WSSV-resistant shrimp ( Penaeus japonicus) by suppression subtractive hybridization. Dev Comp Immunol.

[21] Leu J-H, Chen S-H, Wang Y-B, Chen Y-C, Su S-Y, Lin C-Y (2011). A review of the major penaeid shrimp EST studies and the construction of a shrimp transcriptome database based on the ESTs from four penaeid shrimp. Marine Biotechnol.

[22] Morozova O, Hirst M, Marra MA (2009). Applications of new sequencing technologies for transcriptome analysis. Annu Rev Genomics Hum Genet.

[23] Metzker ML (2010). Sequencing technologies—the next generation. Nat Rev Genet.

[24] Ansorge WJ (2009). Next-generation DNA sequencing techniques. N Biotechnol.

[25] Varshney RK, Nayak SN, May GD, Jackson SA (2009). Next-generation sequencing technologies and their implications for crop genetics and breeding. Trends Biotechnol.

[26] Martin JA, Wang Z (2011). Next-generation transcriptome assembly. Nat Rev Genet.

[27] Reis-Filho JS (2009). Next-generation sequencing. Breast Cancer Res.

[28] Mutz K-O, Heilkenbrinker A, Lönne M, Walter J-G, Stahl F (2013). Transcriptome analysis using next-generation sequencing. Curr Opin Biotechnol.

[29] Asmann YW, Wallace MB, Thompson EA (2008). Transcriptome profiling using next-generation sequencing. Gastroenterology.

[30] Mohd-Shamsudin MI, Kang Y, Lili Z, Tan TT, Kwong QB, Liu H (2013). In-depth tanscriptomic analysis on giant freshwater prawns. PLoS One.

[31] Tiruvayipati S, Bhassu S, Kumar N, Baddam R, Shaik S, Gurindapalli AK (2013). Genome anatomy of the gastrointestinal pathogen, Vibrio parahaemolyticus of crustacean origin. Gut Pathog.

[32] Arockiaraj J, Vanaraja P, Easwvaran S, Singh A, Alinejaid T, Othman RY (2011). Gene profiling and characterization of arginine kinase-1 (MrAK-1) from freshwater giant prawn ( Macrobrachium rosenbergii). Fish Shellfish Immunol.

[33] Arockiaraj J, Easwvaran S, Vanaraja P, Singh A, Othman RY, Bhassu S (2012). Molecular cloning, characterization and gene expression of an antioxidant enzyme catalase ( Mr Cat) from Macrobrachium rosenbergii. Fish Shellfish Immunol.

[34] Arockiaraj J, Easwvaran S, Vanaraja P, Singh A, Othman RY, Bhassu S (2012). Immunological role of thiol-dependent peroxiredoxin gene in Macrobrachium rosenbergii. Fish Shellfish Immunol.

[35] Arockiaraj J, Vanaraja P, Easwvaran S, Singh A, Othman RY, Bhassu S (2011). Bioinformatic characterization and gene expression pattern of apoptosis inhibitor from Macrobrachium rosenbergii challenged with infectious hypodermal and hematopoietic necrosis virus. Fish Shellfish Immunol.

[36] Haas BJ, Papanicolaou A, Yassour M, Grabherr M, Blood PD, Bowden J (2013). De novo transcript sequence reconstruction from RNA-seq using the Trinity platform for reference generation and analysis. Nat Protoc.

[37] Pertea G, Huang X, Liang F, Antonescu V, Sultana R, Karamycheva S (2003). TIGR Gene Indices clustering tools (TGICL): a software system for fast clustering of large EST datasets. Bioinformatics.

[38] Boeckmann B, Bairoch A, Apweiler R, Blatter M-C, Estreicher A, Gasteiger E (2003). The SWISS-PROT protein knowledgebase and its supplement TrEMBL in 2003. Nucleic Acids Res.

[39] Tatusov RL, Galperin MY, Natale DA, Koonin EV (2000). The COG database: a tool for genome-scale analysis of protein functions and evolution. Nucleic Acids Res.

[40] Kanehisa M, Goto S (2000). KEGG: kyoto encyclopedia of genes and genomes. Nucleic Acids Res.

[41] Mount DW (2007). Using the basic local alignment search tool (BLAST). Cold Spring Harb Protoc.

[42] Ashburner M, Ball CA, Blake JA, Botstein D, Butler H, Cherry JM (2000). Gene Ontology: tool for the unification of biology. Nat Genet.

[43] Conesa A, Götz S, García-Gómez JM, Terol J, Talón M, Robles M (2005). Blast2GO: a universal tool for annotation, visualization and analysis in functional genomics research. Bioinformatics.

[44] Iseli C, Jongeneel CV, Bucher P (1999). ESTScan: a program for detecting, evaluating, and reconstructing potential coding regions in EST sequences. ISMB.

[45] Mortazavi A, Williams BA, McCue K, Schaeffer L, Wold B (2008). Mapping and quantifying mammalian transcriptomes by RNA-Seq. Nat Methods.

[46] Reiner A, Yekutieli D, Benjamini Y (2003). Identifying differentially expressed genes using false discovery rate controlling procedures. Bioinformatics.

[47] Schmittgen TD, Livak KJ (2008). Analyzing real-time PCR data by the comparative CT method. Nat Protoc.

[48] Aoki T, Wang H-C, Unajak S, Santos MD, Kondo H, Hirono I (2011). Microarray analyses of shrimp immune responses. Marine Biotechnol.

[49] Gross P, Bartlett T, Browdy C, Chapman R, Warr G (2001). Immune gene discovery by expressed sequence tag analysis of hemocytes and hepatopancreas in the Pacific White Shrimp, Litopenaeus vannamei, and the Atlantic White ShrimpL. setiferus. Dev Comp Immunol.

[50] Supungul P, Klinbunga S, Pichyangkura R, Jitrapakdee S, Hirono I, Aoki T (2002). Identification of immune-related genes in hemocytes of black tiger shrimp (Penaeus monodon). Marine Biotechnol.

[51] Xu J, Ji P, Wang B, Zhao L, Wang J, Zhao Z (2013). Transcriptome Sequencing and Analysis of Wild Amur Ide (Leuciscus waleckii) Inhabiting an Extreme Alkaline-Saline Lake Reveals Insights into Stress Adaptation. PloS One.

[52] Feldmeyer B, Wheat CW, Krezdorn N, Rotter B, Pfenninger M (2011). Short read Illumina data for the de novo assembly of a non-model snail species transcriptome (Radix balthica, Basommatophora, Pulmonata), and a comparison of assembler performance. BMC Genomics.

[53] Garg R, Patel RK, Tyagi AK, Jain M (2011). De novo assembly of chickpea transcriptome using short reads for gene discovery and marker identification. DNA Res.

[54] Zeng D, Chen X, Xie D, Zhao Y, Yang C, Li Y (2013). Transcriptome analysis of pacific white shrimp (Litopenaeus vannamei) hepatopancreas in response to Taura Syndrome Virus (TSV) experimental infection. PLoS One.

[55] Chen X, Zeng D, Chen X, Xie D, Zhao Y, Yang C (2013). Transcriptome analysis of Litopenaeus vannamei in response to white spot syndrome virus infection. PLoS One.

[56] Xue S, Liu Y, Zhang Y, Sun Y, Geng X, Sun J (2013). Sequencing and De Novo Analysis of the Hemocytes Transcriptome in Litopenaeus vannamei Response to White Spot Syndrome Virus Infection. PLoS One.

[57] Akira S, Uematsu S, Takeuchi O (2006). Pathogen recognition and innate immunity. Cell.

[58] Kumar H, Kawai T, Akira S (2011). Pathogen recognition by the innate immune system. Int Rev Immunol.

[59] Liu Y-C, Li F-H, Dong B, Wang B, Luan W, Zhang X-J (2007). Molecular cloning, characterization and expression analysis of a putative C-type lectin (Fclectin) gene in Chinese shrimp Fenneropenaeus chinensis. Mol Immunol.

[60] Ma THT, Tiu SHK, He J-G, Chan S-M (2007). Molecular cloning of a C-type lectin (LvLT) from the shrimp Litopenaeus vannamei: Early gene down-regulation after WSSV infection. Fish Shellfish Immunol.

[61] Sritunyalucksana K, Lee SY, Söderhäll K (2002). A β-1, 3-glucan binding protein from the black tiger shrimp, Penaeus monodon. Dev Comp Immunol.

[62] Marques MRF, Barracco MA (2000). Lectins, as non-self-recognition factors, in crustaceans. Aquaculture.

[63] Adams A (1991). Response of penaeid shrimp to exposure to Vibrio species. Fish Shellfish Immunol.

[64] Sun Y-D, Fu L-D, Jia Y-P, Du X-J, Wang Q, Wang Y-H (2008). A hepatopancreas-specific C-type lectin from the Chinese shrimp Fenneropenaeus chinensis exhibits antimicrobial activity. Mol Immunol.

[65] Cheng W, Liu C-H, Tsai C-H, Chen J-C (2005). Molecular cloning and characterisation of a pattern recognition molecule, lipopolysaccharide-and β-1, 3-glucan binding protein (LGBP) from the white shrimp Litopenaeus vannamei. Fish Shellfish Immunol.

[66] Liu F, Li F, Dong B, Wang X, Xiang J (2009). Molecular cloning and characterisation of a pattern recognition protein, lipopolysaccharide and β-1, 3-glucan binding protein (LGBP) from Chinese shrimp Fenneropenaeus chinensis. Mol Biol Rep.

[67] Soonthornchai W, Rungrassamee W, Karoonuthaisiri N, Jarayabhand P, Klinbunga S, Söderhäll K (2010). Expression of immune-related genes in the digestive organ of shrimp, Penaeus monodon, after an oral infection by Vibrio harveyi. Dev Comp Immunol.

[68] Vargas-Albores F, Yepiz-Plascencia G (2000). Beta glucan binding protein and its role in shrimp immune response. Aquaculture.

[69] Soltanian S, Stuyven E, Cox E, Sorgeloos P, Bossier P (2009). Beta-glucans as immunostimulant in vertebrates and invertebrates. Crit Rev Microbiol.

[70] Amparyup P, Sutthangkul J, Charoensapsri W, Tassanakajon A (2012). Pattern recognition protein binds to lipopolysaccharide and β-1, 3-glucan and activates shrimp prophenoloxidase system. J Biol Chem.

[71] Perazzolo LM, Barracco MA (1997). The prophenoloxidase activating system of the shrimp Penaeus paulensis and associated factors. Dev Comp Immunol.

[72] Sarathi M, Ahmed V, Venkatesan C, Balasubramanian G, Prabavathy J, Hameed A (2007). Comparative study on immune response of Fenneropenaeus indicus to Vibrio alginolyticus and white spot syndrome virus. Aquaculture.

[73] Liu C-H, Yeh S-T, Cheng S-Y, Chen J-C (2004). The immune response of the white shrimp Litopenaeus vannamei and its susceptibility to Vibrio infection in relation with the moult cycle. Fish Shellfish Immunol.

[74] Nayak S, Singh S, Ramaiah N, Sreepada R (2010). Identification of upregulated immune-related genes in Vibrio harveyi challenged Penaeus monodon postlarvae. Fish Shellfish Immunol.

[75] Bachère E (2003). Anti-infectious immune effectors in marine invertebrates: potential tools for disease control in larviculture. Aquaculture.

[76] Rolland J-L, Abdelouahab M, Dupont J, Lefevre F, Bachère E, Romestand B (2010). Stylicins, a new family of antimicrobial peptides from the Pacific blue shrimp Litopenaeus stylirostris. Mol Immunol.

[77] Tassanakajon A, Amparyup P, Somboonwiwat K, Supungul P (2011). Cationic antimicrobial peptides in penaeid shrimp. Marine Biotechnol.

[78] Destoumieux D, Bulet P, Loew D, Van Dorsselaer A, Rodriguez J, Bachère E (1997). Penaeidins, a new family of antimicrobial peptides isolated from the shrimp Penaeus vannamei (Decapoda). J Biol Chem.

[79] Zhang J, Li F, Wang Z, Xiang J (2007). Cloning and recombinant expression of a crustin-like gene from Chinese shrimp, Fenneropenaeus chinensis. J Biotechnol.

[80] De la Vega E, O’Leary NA, Shockey JE, Robalino J, Payne C, Browdy CL (2008). Anti-lipopolysaccharide factor in Litopenaeus vannamei( Lv ALF): a broad spectrum antimicrobial peptide essential for shrimp immunity against bacterial and fungal infection. Mol Immunol.

[81] Burge EJ, Madigan DJ, Burnett LE, Burnett KG (2007). Lysozyme gene expression by hemocytes of Pacific white shrimp, Litopenaeus vannamei, after injection with Vibrio. Fish Shellfish Immunol.

[82] Arockiaraj J, Gnanam AJ, Muthukrishnan D, Gudimella R, Milton J, Singh A (2013). Crustin, a WAP domain containing antimicrobial peptide from freshwater prawn Macrobrachium rosenbergii: Immune characterization. Fish Shellfish Immunol.

[83] Somboonwiwat K, Marcos M, Tassanakajon A, Klinbunga S, Aumelas A, Romestand B (2005). Recombinant expression and anti-microbial activity of anti-lipopolysaccharide factor (ALF) from the black tiger shrimp Penaeus monodon. Dev Comp Immunol.

[84] Arockiaraj J, Avin FA, Vanaraja P, Easwvaran S, Singh A, Othman RY (2012). Immune role of Mr NFκBI-α, an IκB family member characterized in prawn M. rosenbergii. Fish Shellfish Immunol.

[85] Maningas MBB, Kondo H, Hirono I, Saito-Taki T, Aoki T (2008). Essential function of transglutaminase and clotting protein in shrimp immunity. Mol Immunol.

[86] Yeh M-S, Liu C-H, Hung C-W, Cheng W (2009). cDNA cloning, identification, tissue localisation, and transcription profile of a transglutaminase from white shrimp, Litopenaeus vannamei, after infection by Vibrio alginolyticus. Fish Shellfish Immunol.

[87] Liu Y-C, Li F-H, Wang B, Dong B, Zhang Q-L, Luan W (2007). A transglutaminase from Chinese shrimp ( Fenneropenaeus chinensis), full-length cDNA cloning, tissue localization and expression profile after challenge. Fish Shellfish Immunol.

[88] Bandyopadhyay U, Das D, Banerjee RK (1999). Reactive oxygen species: oxidative damage and pathogenesis. Curr Sci.

[89] Yu BP (1994). Cellular defenses against damage from reactive oxygen species. Physiol Rev.

[90] Zhang Q, Li F, Zhang X, Dong B, Zhang J, Xie Y (2008). cDNA cloning, characterization and expression analysis of the antioxidant enzyme gene, catalase, of Chinese shrimp Fenneropenaeus chinensis. Fish Shellfish Immunol.

[91] Zhou J, Wang W-N, Wang A-L, He W-Y, Zhou Q-T, Liu Y (2009). Glutathione S-transferase in the white shrimp Litopenaeus vannamei: characterization and regulation under pH stress. Comp Biochem Physiol C Toxicol Pharmacol.

[92] Cheng W, Tung Y-H, Liu C-H, Chen J-C (2006). Molecular cloning and characterisation of copper/zinc superoxide dismutase (Cu, Zn-SOD) from the giant freshwater prawn Macrobrachium rosenbergii. Fish Shellfish Immunol.

[93] Tian J, Chen J, Jiang D, Liao S, Wang A (2011). Transcriptional regulation of extracellular copper zinc superoxide dismutase from white shrimp Litopenaeus vannamei following Vibrio alginolyticus and WSSV infection. Fish Shellfish Immunol.

[94] Ren Q, Sun R-R, Zhao X-F, Wang J-X (2009). A selenium-dependent glutathione peroxidase (Se-GPx) and two glutathione S-transferases (GSTs) from Chinese shrimp ( Fenneropenaeus chinensis). Comp Biochem Physiol C Toxicol Pharmacol.

[95] Li D-X, Du X-J, Zhao X-F, Wang J-X (2006). Cloning and expression analysis of an o-methyltransferase (OMT) gene from Chinese shrimp, Fenneropenaeus chinensis. Fish Shellfish Immunol.

[96] Li C, Ni D, Song L, Zhao J, Zhang H, Li L (2008). Molecular cloning and characterization of a catalase gene from Zhikong scallop Chlamys farreri. Fish Shellfish Immunol.

[97] He N, Liu H, Xu X (2004). Identification of genes involved in the response of haemocytes of Penaeus japonicus by suppression subtractive hybridization (SSH) following microbial challenge. Fish Shellfish Immunol.

[98] Horváth I, Multhoff G, Sonnleitner A, Vígh L (2008). Membrane-associated stress proteins: more than simply chaperones. Biochim Biophys Acta.

[99] Vabulas RM, Raychaudhuri S, Hayer-Hartl M, Hartl FU (2010). Protein folding in the cytoplasm and the heat shock response. Cold Spring Harb Perspect Biol.

[100] Jiang S, Qiu L, Zhou F, Huang J, Guo Y, Yang K (2009). Molecular cloning and expression analysis of a heat shock protein (Hsp90) gene from black tiger shrimp (Penaeus monodon). Mol Biol Rep.

[101] Lo W-Y, Liu K-F, Liao I-C, Song Y-L (2004). Cloning and molecular characterization of heat shock cognate 70 from tiger shrimp (Penaeus monodon). Cell Stress Chaperones.

[102] Cui Z, Liu Y, Luan W, Li Q, Wu D, Wang S (2010). Molecular cloning and characterization of a heat shock protein 70 gene in swimming crab (Portunus trituberculatus). Fish Shellfish Immunol.

[103] Zhou J, Wang W-N, He W-Y, Zheng Y, Wang L, Xin Y (2010). Expression of HSP60 and HSP70 in white shrimp, Litopenaeus vannamei in response to bacterial challenge. J Invertebr Pathol.

[104] Rungrassamee W, Leelatanawit R, Jiravanichpaisal P, Klinbunga S, Karoonuthaisiri N (2010). Expression and distribution of three heat shock protein genes under heat shock stress and under exposure to Vibrio harveyi in Penaeus monodon. Dev Comp Immunol.

[105] Robalino J, Almeida JS, McKillen D, Colglazier J, Trent HF, Chen YA (2007). Insights into the immune transcriptome of the shrimp Litopenaeus vannamei: tissue-specific expression profiles and transcriptomic responses to immune challenge. Physiol Genomics.

[106] Weinrauch Y, Zychlinsky A (1999). The induction of apoptosis by bacterial pathogens. Annu Rev Microbiol.

[107] Chen Y, Zychlinsky A (1994). Apoptosis induced by bacterial pathogens. Microb Pathog.

[108] Chang C-C, Yeh M-S, Lin H-K, Cheng W (2008). The effect of Vibrio alginolyticus infection on caspase-3 expression and activity in white shrimp Litopenaeus vannamei. Fish Shellfish Immunol.

[109] Fall J, Kono T, Tanekhy M, Itami T, Sakai M (2010). Expression of innate immune-related genes of Kuruma shrimp, Marsupenaeus japonicus, after challenge with Vibrio nigripulchritudo. Afr J Microbiol Res.

[110] Finlay BB, McFadden G (2006). Anti-immunology: evasion of the host immune system by bacterial and viral pathogens. Cell.

[111] Belvin MP, Anderson KV (1996). A conserved signaling pathway: the Drosophila toll-dorsal pathway. Annu Rev Cell Dev Biol.

[112] Yang C, Zhang J, Li F, Ma H, Zhang Q, Jose Priya T (2008). A Toll receptor from Chinese shrimp Fenneropenaeus chinensis is responsive to Vibrio anguillarum infection. Fish Shellfish Immunol.

[113] Li F, Wang D, Li S, Yan H, Zhang J, Wang B (2010). A Dorsal homolog (FcDorsal) in the Chinese shrimp Fenneropenaeus chinensis is responsive to both bacteria and WSSV challenge. Dev Comp Immunol.

[114] Huffman DL, Abrami L, Sasik R, Corbeil J, van der Goot FG, Aroian RV (2004). Mitogen-activated protein kinase pathways defend against bacterial pore-forming toxins. Proc Natl Acad Sci U S A.

[115] Honda T, Ni Y, Miwatani T, Adachi T, Kim J (1992). The thermostable direct hemolysin of Vibrio parahaemolyticus is a pore-forming toxin. Can J Microbiol.

[116] Sun C, Shao H-L, Zhang X-W, Zhao X-F, Wang J-X (2011). Molecular cloning and expression analysis of signal transducer and activator of transcription (STAT) from the Chinese white shrimp Fenneropenaeus chinensis. Mol Biol Rep.

[117] Zong R, Wu W, Xu J, Zhang X (2008). Regulation of phagocytosis against bacterium by Rab GTPase in shrimp Marsupenaeus japonicus. Fish Shellfish Immunol.

[118] Ji P-F, Yao C-L, Wang Z-Y (2011). Two types of calmodulin play different roles in Pacific white shrimp ( Litopenaeus vannamei) defenses against Vibrio parahaemolyticus and WSSV infection. Fish Shellfish Immunol.

[119] Maiti B, Khushiramani R, Tyagi A, Karunasagar I, Karunasagar I (2010). Recombinant ferritin protein protects Penaeus monodon infected by pathogenic Vibrio harveyi. Dis Aquat Organ.

[120] YAN F, ZHANG Y-l, LUO H-q, HU Z, HUANG T-w, YE X-q (2008). The phenoloxidase activity of hemocyanin from white leg shrimp Litopenaeus vannamei. Fisheries Sci.

[121] Zhang X, Huang C, Qin Q (2004). Antiviral properties of hemocyanin isolated from shrimp Penaeus monodon. Antiviral Res.

