# Supplementary figures and images for: RNA-seq analysis of Macrobrachium rosenbergii hepatopancreas in response to Vibrio parahaemolyticus infection
Source: Gut Pathog. 2015 Mar 14;7:6. doi: 10.1186/s13099-015-0052-6 (PMC4411767; doi:10.1186/s13099-015-0052-6)

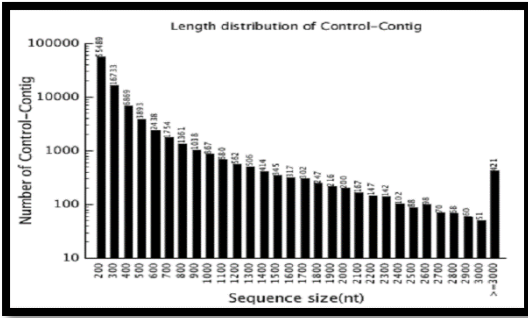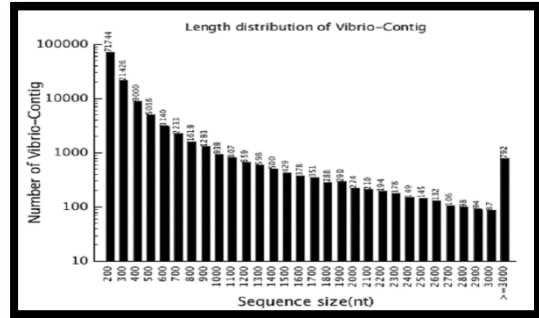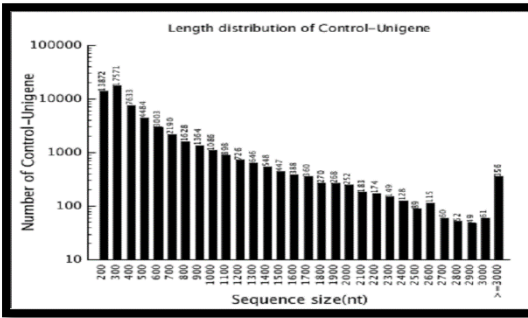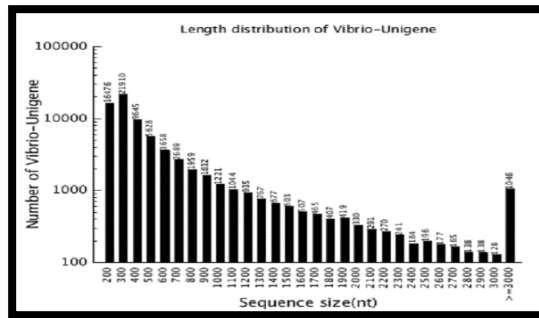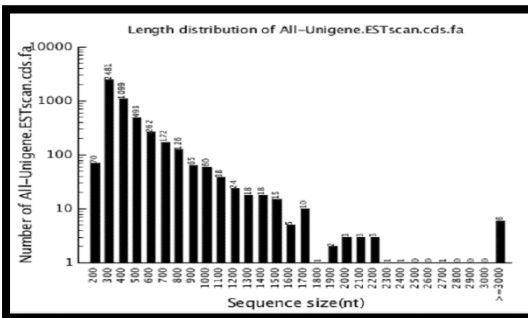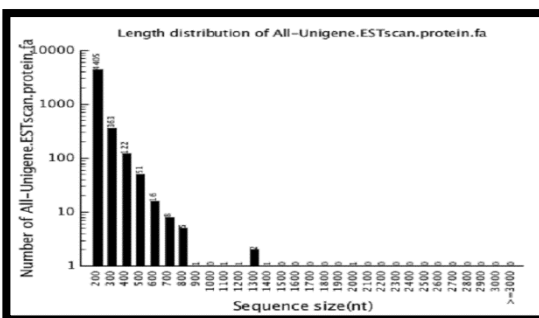

Supplement: Additional file 2: Figure S1. — Overview of the control and V. parahaemolyticus infected transcriptome assembly. (A) The size distribution of the contigs obtained from our denovo assembly of high-quality clean reads. (B) The size distribution of the unigenes produced from further assembly of contigs (i.e., contig joining, gap filling, and scaffold clustering). (C) Size distributions of the coding sequences (CDS) and identified proteins and (D) Size distributions of the ESTs and proteins obtained from the ESTScan results. For unigene CDS that had no hits in the databases (Nr, SwissProt, KEGG and COG), the BLAST results were subjected to ESTScans and then translated into peptide sequences. [file 13099_2015_52_MOESM2_ESM.pdf]
